# Spinal cord stimulation for predominant low back pain in failed back surgery syndrome: study protocol for an international multicenter randomized controlled trial (PROMISE study)

**DOI:** 10.1186/1745-6215-14-376

**Published:** 2013-11-07

**Authors:** Philippe Rigoard, Mehul J Desai, Richard B North, Rod S Taylor, Lieven Annemans, Christine Greening, Ye Tan, Carine Van den Abeele, Jane Shipley, Krishna Kumar

**Affiliations:** 1 Department of Neurosurgery, Poitiers University Hospital, Poitiers, France; 2 Metro Orthopedics & Sports Therapy, 8401 Colesville Road #50, Silver Spring, MD 20910, USA; 3 The Neuromodulaton Foundation, Inc., 10807 Falls Road, #379, Brooklandville, MD 21022, USA; 4 Institute of Health Research, Exeter Medical School, University of Exeter, Veysey Building, Salmon Pool Lane, Exeter EX2 4SG, UK; 5 Ghent University, Department of Public Health, De Pintelaan 185, Ghent 9000, Belgium; 6 Department of Clinical Research, Medtronic, Inc., Rice Creek East, 7000 Central Avenue NE, Minneapolis, MN 55432-3568, USA; 7 Department of Clinical Research, Medtronic International Trading Sàrl, Route du Molliau 31, Case Postale 1131, Tolochenaz, Switzerland; 8 Department of Neurosurgery, Regina General Hospital, University of Saskatchewan, Regina, SK, Canada

**Keywords:** Low back pain, Neuropathic pain, Failed back surgery syndrome, Spinal cord stimulation, Randomized controlled trial

## Abstract

**Background:**

Although results of case series support the use of spinal cord stimulation in failed back surgery syndrome patients with predominant low back pain, no confirmatory randomized controlled trial has been undertaken in this patient group to date. PROMISE is a multicenter, prospective, randomized, open-label, parallel-group study designed to compare the clinical effectiveness of spinal cord stimulation plus optimal medical management with optimal medical management alone in patients with failed back surgery syndrome and predominant low back pain.

**Method/Design:**

Patients will be recruited in approximately 30 centers across Canada, Europe, and the United States. Eligible patients with low back pain exceeding leg pain and an average Numeric Pain Rating Scale score ≥5 for low back pain will be randomized 1:1 to spinal cord stimulation plus optimal medical management or to optimal medical management alone. The investigators will tailor individual optimal medical management treatment plans to their patients. Excluded from study treatments are intrathecal drug delivery, peripheral nerve stimulation, back surgery related to the original back pain complaint, and experimental therapies. Patients randomized to the spinal cord stimulation group will undergo trial stimulation, and if they achieve adequate low back pain relief a neurostimulation system using the Specify® 5-6-5 multi-column lead (Medtronic Inc., Minneapolis, MN, USA) will be implanted to capture low back pain preferentially in these patients. Outcome assessment will occur at baseline (pre-randomization) and at 1, 3, 6, 9, 12, 18, and 24 months post randomization. After the 6-month visit, patients can change treatment to that received by the other randomized group. The primary outcome is the proportion of patients with ≥50% reduction in low back pain at the 6-month visit. Additional outcomes include changes in low back and leg pain, functional disability, health-related quality of life, return to work, healthcare utilization including medication usage, and patient satisfaction. Data on adverse events will be collected. The primary analysis will follow the intention-to-treat principle. Healthcare use data will be used to assess costs and long-term cost-effectiveness.

**Discussion:**

Recruitment began in January 2013 and will continue until 2016.

**Trial registration:**

Clinicaltrials.gov: NCT01697358 (http://www.clinicaltrials.gov)

## Background

Following lumbosacral spine surgery, between 5 and 50% of patients suffer from chronic back and/or leg pain, commonly known as failed back surgery syndrome (FBSS) [1,2] and characterized by disabling neuropathic radicular leg pain with or without low back pain, which might have mixed neuropathic and nociceptive pain components [3]. FBSS can be disabling and can have a negative impact on a patient’s health-related quality of life, well-being, and productivity [4,5].

Treatment of FBSS pain by further operations or medical management is often unsuccessful and places a heavy economic financial burden on healthcare systems [2,3]. In contrast, randomized controlled trials (RCTs) have shown spinal cord stimulation (SCS) to be a clinically effective and cost-effective adjunct to medical management [6-8] or alternative to a further operation [9,10] in patients with FBSS characterized by leg pain exceeding or equaling axial low back pain.

The effective targeting of the back pain component of FBSS remains a challenge [11-13]. Ongoing development of SCS leads and electrode arrays has included creation of an insulated paddle with a multicolumn design and a greater number of contacts than was formerly available: all of these features improve low back coverage [14-16]. A pilot study has demonstrated the efficacy of a multicolumn SCS lead configuration for the treatment of the axial back pain component of FBSS [17]. RCT evidence is needed to confirm the efficacy of the use of multicolumn SCS in patients with FBSS characterized by predominant back pain.

### Aims and objectives

The PROMISE study is a RCT comparing the clinical effectiveness of SCS using the Specify® 5-6-5 surgical lead (Medtronic, Inc., Minneapolis, MN, USA) plus optimal medical management (OMM) versus OMM alone in patients suffering from predominant low back pain due to FBSS. After the 6-month visit, patients can change treatment to that received by the other randomized group. All patients will be followed for 24 months post randomization.

The primary objective is to determine the difference in the proportion of patients receiving SCS plus OMM versus the proportion receiving OMM alone who have ≥50% reduction in back pain intensity, as measured by the Numeric Pain Rating Scale (NPRS), from baseline to 6 months. Secondary objectives are to compare changes in the following outcomes from baseline to 6 months between the two groups: low back pain intensity, as measured by the NPRS [18]; leg pain intensity, as measured by the NPRS [18]; functional disability, as measured by the Oswestry Disability Index [19]; and quality of life, as measured by the Short-Form Health Survey, version 2 physical component summary score [20]. Additional objectives include the comparison of other patient-related outcomes and healthcare utilization including medication usage (see below) at 6 months, to assess within-patient changes (that is, compared with pre-randomization) in outcomes from 6 to 24 months post randomization, to characterize all SCS-related and non-SCS-related adverse events, and to assess the costs and cost-effectiveness of SCS plus OMM versus OMM alone.

## Methods/Design

This protocol is reported in accord with the SPIRIT 2013 guidance for protocols of clinical trials [21].

### Design and setting

This multicenter study has an open-label, randomized, parallel-group design. Patients will be randomized 1:1 to SCS using the Specify® 5-6-5 surgical lead (Medtronic, Inc.) plus OMM (SCS group) or to OMM alone (OMM group) and followed for 6 months (Period I, randomized comparative phase). After completion of the 6-month (Period I) outcome assessment, patients will be systematically reminded (through electronic tablets used for questionnaire completion) that they have completed the randomized period of the study and can change treatment to that received by the other randomized group. Reasons for treatment switching will be recorded. Patients will be followed to 24 months post randomization (Period II, long-term observational follow-up phase).

**Figure 1:**
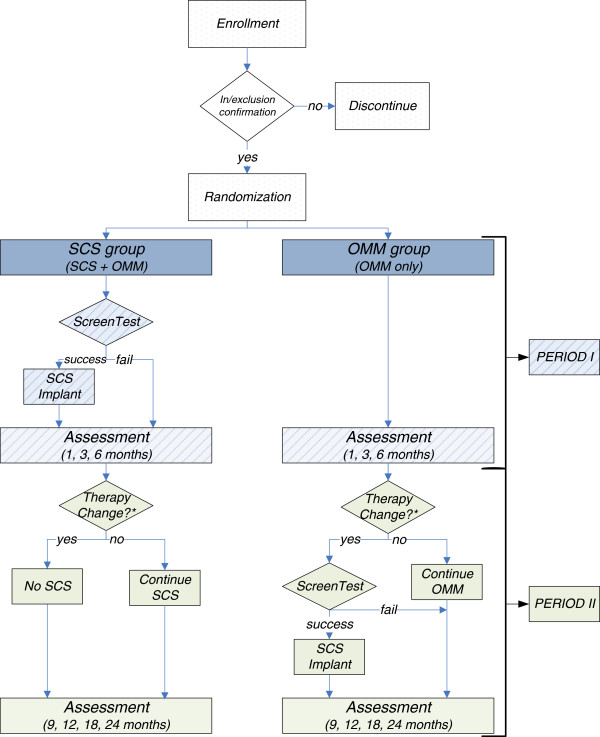
**PROMISE trial patient flow.** *Therapy change may occur at any time post 6-month visit. OMM, optimal medical management; SCS, spinal cord stimulation.

The trial will be conducted at approximately 30 investigative sites globally, with approximately 15 to 20 sites in the USA and 10 to 15 sites in Europe and Canada. Additional locations may be added during the course of the study. No single center can exceed 10% of all randomized patients. The study design is summarized in Figure 1.

### Selection of patients

The study population comprises patients suffering from chronic low back (predominant) and leg pain due to FBSS. A patient must meet all of the inclusion criteria and none of the exclusion criteria to be eligible for the study.

#### ***Inclusion criteria***

The subject is a candidate for SCS with the multicolumn Specify® 5-6-5 surgical lead (see Additional file 1); has FBSS and does not require further surgery (for the purposes of this study, FBSS is defined as persistent or recurrent low back and leg pain of at least 6 months duration, following at least one decompression and/or fusion procedure); presents average low back pain ≥5 and that is greater than leg pain as assessed by the baseline NPRS; and has persistent low back and leg pain despite other treatment modalities (pharmacological, surgical, physical, or psychological therapies) that have been tried and did not prove satisfactory, are unsuitable, or are contraindicated for the subject.

#### ***Exclusion criteria***

The subject is being treated or has been treated with SCS, subcutaneous or peripheral nerve stimulation, being treated with an intrathecal drug delivery system or requires back surgery at the location related to his/her original back pain complaint or experimental therapies; had most recent back surgery less than 6 months ago; has low back pain only (no leg pain) as assessed by the baseline NPRS; is suspected by the investigator of substance abuse that might confound the study results; has unresolved major issues of secondary gain, as determined by the investigator; exhibits major psychiatric morbidity, untreated or refractory to treatment as determined by the investigator; has consistent severe pain (that is, 10 out of 10) without fluctuation, which might confound the results of this study; has radiographic evidence of instability requiring fusion; has pain relieved completely by recumbency (mechanical pain); has a serious neurologic deficit; has a history of coagulation disorder, lupus erythematosus, diabetic neuropathy, rheumatoid arthritis, or ankylosing spondylitis; has calcific arachnoiditis; has severe thoracic stenosis; has life expectancy < 24 months beyond study enrolment; is <18 years of age; is pregnant or planning to become pregnant during the course of the study; is enrolled in or plans to enroll in any study that might confound the results of this study; would be unable to operate the SCS equipment, based on the opinion of the principal or sub-investigator; is unwilling to be treated with SCS, attend visits as scheduled, and/or comply with study requirements; is unable to undergo study assessments or complete questionnaires independently (for example, is illiterate); and is a member of a vulnerable population.

A screening log will be kept at each site to identify potential subjects. All FBSS patients identified through standard clinical practice at the site (for example, call log, chart review, scheduled visits, and referrals) will be listed on the screening log. Potentially eligible subjects will be provided with detailed information about the study including a description of SCS and of OMM.

A patient is considered enrolled in the study upon completion of the informed consent process. Enrolled subjects will be taught how to complete a pain diary between the enrolment visit and the randomization visit. The subject will record his/her low back and leg pain scores during a 7-day pain diary period prior to randomization. Subjects will not be told what confirmatory pain scores are required for study inclusion. The inclusion criteria of average NPRS score to assess low back and leg pain will be evaluated after the subject completes the pain diary. Candidacy for SCS with the Specify® 5-6-5 implant will also be confirmed based on appropriate imaging according to usual practice. In addition, where a psychological evaluation (or other evaluation) is standard of care and/or required, the evaluation must take place prior to randomization. The principal investigator will document whether the patient has received the required evaluation and the results of that evaluation. FBSS diagnosis will also be confirmed based on appropriate imaging according to usual practice.

The principal investigator or his/her designee will review the diary to confirm that the pain intensity scores and pain location meet the inclusion/exclusion criteria (see above). In addition, the principal investigator will document that the patient is a candidate for implantation of a Specify® 5-6-5 SCS system. Upon confirmation of eligibility, OMM treatment planning and completion of the questionnaires, subjects will be randomized to one of the two treatment groups. Subjects who do not complete or meet preliminary eligibility requirements will not be randomized and will be exited from the study.

### Interventions

Pain treatment will be evaluated, and medical management of patient’s pain will be optimized in both arms. As part of the confirmation of eligibility (prior to randomization), the investigator and subject will determine an individual OMM treatment plan, which should include non-investigational pharmacologic agents (for example, tricyclic antidepressants, opioid analgesics or tramadol, antiepileptics, or lidocaine) and/or interventional therapies (for example, therapeutic injections, radiofrequency, acupuncture, functional restoration, physical therapy, and psychological interventions, such as cognitive behavioral therapy) as appropriate. The following treatments are excluded from OMM: intrathecal drug delivery, peripheral nerve stimulation (not an approved indication in the United States), back surgery at the location related to the patient’s original back pain complaint, and experimental therapies. Data regarding pain treatments implemented during the study will be collected to reveal how medical management was optimized. After randomization, as well as at all scheduled follow-up visits, the subject and physician will further discuss OMM to determine the best course of continued action.

In addition to OMM, patients randomized to the SCS arm will undergo an SCS screening test (3-day minimum). The screening test may be conducted with the Specify® 5-6-5 surgical lead or with a percutaneous lead(s). If successful, a SCS system will be implanted. A screening test will be determined to be successful if the subject finds the feeling of paresthesia acceptable and has adequate low back pain relief with usual activity and appropriate analgesia as assessed by the physician. Physicians can consider a conducting second screening test with the Specify® 5-6-5 lead if a screening test with a percutaneous lead led to inadequate paresthesia coverage of low back pain and/or painful extraneous stimulation (for example, chest wall pain, pressure or sharp mid-back pain). The final system implanted will consist of a Medtronic pulse generator (rechargeable or primary cell) and a Specify® 5-6-5 surgical lead. Subjects should receive their permanent implant within 60 calendar days from randomization. They will be programmed to their optimal programming parameters and will be able to adjust their stimulation with the patient programmer, within the settings programmed in the clinic. Subjects will be provided with a patient programmer manual and will be instructed on the proper use and handling of the patient programmer. Subjects who fail the screening test (and do not proceed to implant) will be followed in the study according to the principles of intention-to-treat analysis.

### Assessments

Outcome measures selected for this trial are based on a review of the previous RCTs of SCS and a consideration of IMMPACT recommendations [18].

The day of randomization is day 0 for the study periods. Subjects will be assessed prior to randomization (baseline) and at 1-month, 3-month, 6-month, 9-month, 12-month, 18-month and 24-month follow-up visits. Visit windows for Period I and Period II will remain constant based on the original date from the randomization visit, regardless of whether any change in therapy occurs. Assessments will be performed by appropriately trained and delegated study staff according to the usual practices of the site. Electronic case report forms will be used for this study with patients completing the study questionnaires confidentially on electronic tablets, and these data will be uploaded to a web-based server. Table 1 provides a summary of the data collection process and timing.

**Table 1:** PROMISE study – summary of data collection

**Study procedure**	**Enrollment**	**Randomization**	**Screening test**	**Implant**	**1 month**^**a**^	**3 months**^**b**^	**6 months**^**b**^	**9 months**^**b**^	**12 months**^**b**^	**18 months**^**b**^	**24 months**^**b**^	**Unscheduled**	**Early discontinue**^**c**^
Time window (days)	-90 to 0	0	0 to 57	3 to 60	10 to 67	68 to 137	138 to 228	229 to 319	320 to 455	456 to 637	638 to 789	NA	NA
Informed consent	**X**												
Demographics (age, gender)	**X**												
Medical history	**X**												
Inclusion/exclusion criteria	**X**	**X**											
Pain location	**X**	**X**											
Pain diary (complete prior to visit)		**X**			**X**	**X**	**X**	**X**	**X**	**X**	**X**		**X**
OMM treatment plan		**X**			**X**	**X**	**X**	**X**	**X**	**X**	**X**		
Questionnaires (ODI, SF-36, EQ-5D^d^, Pittsburgh Sleep Quality Index, employment status, pain and/or paresthesia map)		**X**				**X**	**X**	**X**	**X**	**X**	**X**		**X**
Randomization assignment		**X**											
Neuropathic pain (DN4)		**X**											
PGIC, Patient satisfaction with therapy						**X**	**X**	**X**	**X**	**X**	**X**		**X**
Nondrug pain treatment and other healthcare use assessment		**X**	**X**	**X**	**X**	**X**	**X**	**X**	**X**	**X**	**X**		**X**
Payer information (US only)	**X**								**X**		**X**		
Medication assessment		**X**	**X**	**X**	**X**	**X**	**X**	**X**	**X**	**X**	**X**		**X**
Event assessment^d^		**X**	**X**	**X**	**X**	**X**	**X**	**X**	**X**	**X**	**X**	**X**	**X**
Screening test assessment			**X**										
Implant information			**X**	**X**									
Request for change in randomized therapy							**X**	**X**	**X**	**X**	**X**	**X**^**e**^	
SCS therapy thresholds							**X**						
Device interrogation (initial and final)				**X**^**f**^	**X**	**X**	**X**	**X**	**X**	**X**	**X**	**X**	**X**

DN4, Douleur neuropathique en 4 questions; ODI, Oswestry Disability Index; OMM, optimal medical management; PGIC, Patient Global Impression of Change; SF-36, Short-Form 36; SCS, spinal cord stimulation.

^a^For subjects randomized to SCS, the 1-month visit should occur a minimum of 7 days after implant (or screening test if the test was unsuccessful).

^**b**^Visits should occur within the window but at least 2 weeks after the previous visit.

^c^Early discontinuation, collect data if possible.

^d^When possible, collect additional EuroQoL (EQ-5D) at time of event if device-related event requiring surgical intervention or hospitalization occurred.

^e^Only after 6 months.

^f^No initial interrogation at implant, final only.

#### ***Primary outcome***

The primary outcome is the proportion of subjects with ≥50% reduction in low back pain intensity measured using the NPRS at 6 months [18]. Pain relief will be assessed as a reduction of the 11-point NPRS. Patients will record their low back and leg pain using a (paper) pain diary two times per day, once in the morning and once in the evening, for a 7-day period within 2 weeks prior to the randomization visit and subsequent scheduled study visits. For the initial baseline pain diary, patients are required to complete at least 5 of 7 days in their entirety, or they will not proceed to randomization. The proportion of subjects in each group with ≥50% reduction in average low back pain score at the end of Period I compared with baseline will be calculated.

#### ***Secondary outcomes***

Secondary outcomes are: Back and leg NPRS score [18]; Oswestry Disability Index version 2 [19]; and Physical Component Summary score on the Short-Form 36 [20].

Other outcomes include: Mental Component Summary score on Short-Form 36 [20]; EuroQoL 5D-5 L (EQ-5D-5 L) [22]; Pittsburgh Sleep Quality Index [23]; healthcare use related to the pain condition and to its treatment (for example, managing adverse events, interventions, investigations, drugs, length and number of inpatient hospitalizations, and number of emergency room, office, and other healthcare-related visits) [24]; employment status ('What is your current employment status?’ , 'If out of work: what is the main reason you are out of work?’); patient satisfaction ('Would you recommend this therapy to patients suffering from pain similar to yours?’ , 'Overall how satisfied or unsatisfied are you with this therapy?’); Patient Global Impression of Change version 2 [25]; programming parameters and paresthesia coverage; and adverse events (adverse events and device deficiencies documentation will include date of adverse event or device deficiency, diagnosis and description of the event, assessment of seriousness of the event, treatment of the event, outcome or resolution and date of the event, and relationship of the event to the device or to OMM – in addition to the standard questionnaire data collection schedule, the EQ-5D-5 L will be collected, when possible, whenever a device-related adverse event occurs that might require a surgical intervention or hospitalization).

#### ***Process measures***

At the screening and baseline visits, the following additional information will be collected: subject demographics (for example, age, gender) and a neuropathic pain assessment by clinician-administered diagnostic questionnaire (Douleur Neuropathique en 4 questions) to discriminate neuropathic pain components of low back pain [26]. Subjects who proceed to device implantation will have the following information collected: pain/paresthesia map, parameter settings, electrode location, and device implant information (that is, model, serial number). Patients receiving SCS therapy will have a device interrogation at each visit, and at only the 6-month visit data will be gathered on the parameters at which SCS paresthesia is perceived, is comfortable, and becomes uncomfortable. Unscheduled patient visits could occur between scheduled study follow-up visits due to patient discontinuation from the study or device reprogramming and management of any complications.

### Sample size and power calculations

Based on the results of the PROCESS study [8], with the assumption that OMM will provide slightly better low back pain relief and a conservative estimate using intention-to-treat analysis, the sample size calculation assumed a 20% between-group difference in the responder rate, which is defined as patients achieving ≥50% low back pain relief, with a 40% response rate for the SCS group and 20% for OMM group. The sample size was calculated using EAST version 5.4, the module Binomial Superiority Trials: two-sample test – difference of proportions, with the following sample size parameters: control group proportion response = 0.2, difference in proportion = 0.2, significance level = 0.05 (two-sided), power = 90%, assigned fraction = 0.5 (randomization ratio = 1:1), unpooled standard deviation and continuity correction for the standardized test statistics; one unequally spaced interim look at 66% (2/3) of the sample for sample size re-estimation, and the Lan–DeMets with O’Brien–Fleming boundary to reject the null hypothesis. These resulted in a total required sample size of 212 randomized subjects (106 subjects per treatment group). To reduce the possibility that one site with atypical results will overly influence combined results, no site can randomize more than 25 of 212 subjects without prior approval. If the sample size is extended, no site can randomize more than 10% of the patients. Assuming 30% pre-randomization attrition, up to 300 subjects may be enrolled.

A single interim analysis is planned after 140 randomized subjects reach the end of Period I. This analysis will provide data to support a decision to re-estimate the sample size if necessary in order to achieve conditional statistical power of at least 90%. For planning and simulation purposes, a ceiling of 400 randomized patients is imposed for the final analysis.

Enrolment, randomization, and follow-up will continue during the interim analysis. The interim analysis will be conducted by an independent statistician, and one of the following possible outcomes will be communicated to the trial sponsor: continue the study with a total of 212 subjects (implies that ≥90% conditional power will be achieved with *n* = 212); or increase the sample size to a specified size in order to achieve ≥90% conditional power to achieve superiority based on the observed interim results.

If the sample size must be increased, the sponsor will determine whether or not to continue the study. It is not anticipated that the sample size will be reduced below 212.

### Procedures to minimize bias

To minimize selection bias, randomization numbers will be assigned in strict sequence; that is, when a subject is confirmed as eligible for randomization, the next unassigned randomization number in sequence will be given. Permuted blocks will be used to generate the randomization assignments, in order to keep the balance of patients receiving each treatment assignment and help prevent the next treatment group from being discerned by site personnel. Randomization allocation will be concealed from the clinician and patient, using a centralized automatic web-based data management system.

Regarding assessment bias, due to the nature of the treatments, the study cannot be blinded (the form of SCS used in this trial requires paresthesia); however, to minimize potential assessment bias, questionnaires will be completed by patients without study staff consultation or visibility, using a secured electronic tablet.

To minimize attrition bias, if a subject misses a study visit the site’s study staff will make at least three documented attempts to bring that subject in for a study visit. The numbers and reasons for dropouts and losses to follow-up will be reported for each arm of the study.

To quantify potential performance bias, the level of OMM treatment and the addition/removal of SCS therapy for all participants at each follow-up to 24 months will be recorded. Additionally, we will compare changes in pain medication use and other healthcare utilization from baseline to the end of Period I for subjects in the SCS group with those in the OMM group. Any imbalance in OMM therapy between study arms will be considered in interpreting between-group outcome differences.

### Data analysis

#### ***Statistical analysis***

For primary and secondary objectives, the primary analysis method will follow the intention-to-treat principle supported by completers’ and as-treated analyses. The following definitions will be applied: intention-to-treat analysis, between-group comparison based on random allocation of all subjects at the 6-month visit; completers’ analysis, between-group comparison based on random allocation of subjects with complete data at the 6-month visit; and as-treated analysis, between-group analysis based on the treatment subjects with complete data at the 6-month visit received at the 6-month visit.

The primary objective will be compared by *Z*-test using unpooled standard deviation with continuity correction, and a logistic regression model will be used for exploratory analysis. Secondary objectives will be assessed using linear regression models. To assess potential differences in treatment effect across sites, a treatment-by-site interaction term will be added to models. If the treatment-by-site interaction term approaches significance (defined as <0.10, as recommended by Fleiss [27]), the term will remain in the final model along with the term for site. In this case, the results for the objective will also be tabulated by site. Factors that might explain differences among sites will be explored.

There is no compelling evidence of patient-related (for example, age, sex) factors that predict the outcome of SCS; however, in order to identify baseline characteristics that might have predictive effects on outcomes, exploratory analysis with regression models may be conducted including baseline covariates. For the analysis of secondary objectives, baseline assessments for pain score and the Oswestry Disability Index will be included in the regression models as covariates.

Missing data are a potential source of bias. A rigorous study design and execution will help prevent the incidence of missing data from occurring. For the primary objective, patients with missing data at the end of Period I will be treated as nonresponders (failures). With this approach, we assume that nonresponders in both treatment groups have no change in their outcomes compared with baseline. This method has been chosen over more complex imputation methods, such as multiple imputation, because it has been empirically demonstrated in trials of chronic pain to produce conservative treatment effects [28]. For the secondary study objectives, no change from baseline will be assumed for patients with missing data. For completers’ and as-treated supporting analyses, only completers will be included in the analysis.

Period II is the long-term follow-up phase, which includes the possibility for patients to change from their original treatment assignment to the alternative treatment. The objectives of Period II are to compare within-patient changes (that is, baseline versus follow-up) in each group. Because the study design allows patients to change from the randomized treatment group to the alternative treatment after 6 months of follow-up, the analysis of objectives in Period II will be based on the treatment patients actually received and not on the randomization assignment. Only completers will be included.

Unadjusted *P* values will be reported. To maintain an overall type I error rate at 0.05, a fixed-sequence method for the multiplicity adjustment of hierarchical endpoints will be used to test the primary and secondary objectives. No multiplicity adjustment will be performed for evaluations of the additional study objectives. A validated statistical software package will be used for the analyses of the study results (for example, SAS, SPLUS). These analyses are detailed in a statistical analysis plan.

#### ***Economic evaluation***

Economic analyses consider the costs and effectiveness of treatments. Costs will be calculated by multiplying units of healthcare use used by the cost of each unit. Effectiveness will be expressed as quality-adjusted life-years, with these data derived from patients’ responses to the EQ-5D-5 L.

A within-trial cost-consequence analysis will include a disaggregated report of costs and quality-adjusted life-years and of other health outcomes observed in the two groups at 6, 12, and 24 months [29]. Cost utility analysis modeling, extrapolated over a longer time horizon (that is, 15 years), will estimate the incremental cost per quality-adjusted life-year ratio for SCS and OMM. These analyses will be detailed later in a health economic analysis plan.

### Ethics and governance

Each investigation site’s Ethics Committee/Institutional Review Board (EC/IRB) will be required to approve the clinical investigational plan (CIP), the written patient information and consent form, any other written information to be provided to the patients, and, if applicable, product labeling and materials used to recruit patients. EC/IRB approval of the study must be provided in the form of a letter before commencement of the study at the investigation site. Site EC/IRB approvals at the time of submission (3 July 2013) of this protocol are listed in Additional file 2.

Each principal investigator will ensure that no study-related activities occur prior to EC/IRB approval and will provide adequate oversight to ensure that the study is conducted in accordance with the outlined standards. Study conduct will be in accordance with the ethical principles that have their origin in the latest version of the World Medical Association Declaration of Helsinki – Ethical Principles for Medical Research Involving Human Subjects (October 2008), Clinical Investigation of Medical Devices for Human Subjects – Good Clinical Practice (International Organization for Standardization 14155:2011) [30] and the laws and regulations of the countries in which the study is conducted (including data protection laws) and the requirements of the study CIP.

Prior to site initiation or subsequent involvement in study activities, the sponsor will provide study training relevant and pertinent to the personnel conducting study activities, including investigator responsibilities and device training (for example, study recommendations for implant procedures and programming, and the requirements of the CIP, informed consent process, and case report forms). Study-specific training will be documented prior to site initiation.

Monitoring visits will be conducted periodically according to the requirements of the study monitoring plan. The monitoring plan will also define source data to be directly recorded on the electronic clinical report form. This information will be provided to the site during site initiation. After site initiation and training, monitoring visits will be conducted periodically to assess site study progress, the investigator’s adherence to the CIP, regulatory compliance (including but not limited to EC/IRB approval and review of the study), maintenance of records and reports, and review of source documents against patient electronic clinical report forms. Monitors will facilitate site regulatory and study compliance by identifying any findings of noncompliance and communicating those findings along with recommendations for preventative/corrective actions to site personnel. Monitors may work with study personnel to determine and recommend appropriate corrective action(s) and to identify trends within the study or at a particular site.

### Trial committees

The PROMISE Steering Committee consists of renowned specialists with expertise in the area of pain management and FBSS surgical procedures (PR, MJD, RBN, KK), statistics (RST), and health economics (LA). The committee will meet periodically to advise on trial design and to monitor enrolment, clinical site progress, and CIP compliance. The Steering Committee also meets with representatives of the sponsor (CG, CVdA) and is supported by a Medtronic statistician (YT) and by a consultant (JS). A data-monitoring committee will not be established for the study because no interventions intended to prolong life or reduce risk of a major adverse health outcome (for example, a cardiovascular event) are evaluated for which favorable or unfavorable study results might suggest study termination, and nor are there safety concerns suggesting the need for a data-monitoring committee.

A publication committee will develop a publication strategy and oversee the development and review of publications related to the study. The publication committee will include members of the steering committee (PR, MJD, RBN, KK, RST) and sponsor representatives (CG, YT). The publication committee will be responsible for overseeing and ensuring that the publication strategy is executed according to the established plan.

An independent clinical events committee consisting of a minimum of three independent physicians will review adverse events to determine any relationship to the SCS therapy.

## Discussion

The two published RCTs on the effectiveness of SCS have explicitly included only subjects with leg pain predominant or equaling low back pain [6,10]. Few of the numerous case series of SCS for FBSS have considered a predominant low back pain population [31].

The PROMISE trial seeks to provide clinical evidence on pain scores, function, health-related quality of life, and cost-effectiveness for FBSS patients with predominant low back pain being treated with SCS using the Specify® 5-6-5 multi-column lead.

## Trial status

The PROMISE trial began patient recruitment in January 2013. Recruitment is expected to close in 2016. It is anticipated that primary endpoint findings will be available in 2017.

## Abbreviations

CIP: Clinical investigational plan; EC/IRB: Ethics Committee/Institutional Review Board; EQ-5D-5 L: EuroQoL; FBSS: Failed back surgery syndrome; NPRS: Numeric Pain Rating Scale; OMM: Optimal medical management; RCT: Randomized controlled trial; SCS: Spinal cord stimulation.

## Competing interests

PR has received consultancy fees from Medtronic and honoraria for medical training from St Jude Medical. MJD has received consultancy fees from Medtronic Inc. and Kimberly-Clark. RBN’s former employers, Johns Hopkins University and Sinai Hospital, and the nonprofit Neuromodulation Foundation, of which he is an unpaid officer, have received funds from manufacturers of SCS equipment (Boston Scientific, Medtronic, St. Jude Medical); he has equity/consulting interest in Algostim. RST has received consultancy fees from Medtronic. LA has received consultancy fees from Medtronic and from Nevro Inc., and honoraria for lectures from Medtronic. CG, CVdA, and YT are full-time employees of Medtronic. JS’s employer (The Neuromodulation Foundation) has consulting agreements with and has received grants from companies that manufacture SCS equipment. KK works as a consultant to Medtronic Inc. and Boston Scientific and has received research grants from both these companies.

## Authors’ contributions

PR is the overall study principal investigator. PR, MJD, RBN, RST, LA, CG, YT, CVdA, JS, and KK all participated in the study conception and design, contributed to the writing of the study protocol, drafting, and editing of this manuscript and read and approved the final manuscript.

## Supplementary Material

Additional file 1 **Is the Specify® 5-6-5 surgical lead technical description.** Click here for file

Additional file 2 Is a table presenting the Site Ethics/IRB approvals (as of 3 July 2013). Click here for file
