# Supplementary material for: Spinal cord stimulation for predominant low back pain in failed back surgery syndrome: study protocol for an international multicenter randomized controlled trial (PROMISE study)
Source: Trials. 2013 Nov 7;14:376. doi: 10.1186/1745-6215-14-376 (PMC4226255; doi:10.1186/1745-6215-14-376)
Supplement: Additional file 1 — Is the Specify® 5-6-5 surgical lead technical description. [file 1745-6215-14-376-S1.docx]

**Additional file 1.**

**Specify® 5-6-5 surgical lead technical description**

The Specify**®** 5-6-5 Model 39565 surgical lead (Medtronic, Inc., Minneapolis, MN, USA) has a staggered 3-column, 16-contact paddle on the distal end. These contacts along the lead can be neutral or are programmed to function as cathodes or anodes to direct the flow of current through the surrounding tissue. The two proximal ends fit into eight-conductor connectors (i.e., two 1x 8 extensions or a implanted Neuromodulation system, Medtronic, Inc., Minneapolis, MN, USA)..). One lead body has a white marker band to indicate contacts 0 to 7. The lead comes in 65 cm and 30 cm lengths and can be connected to the implanted neuromodulation system directly or with extensions if needed.


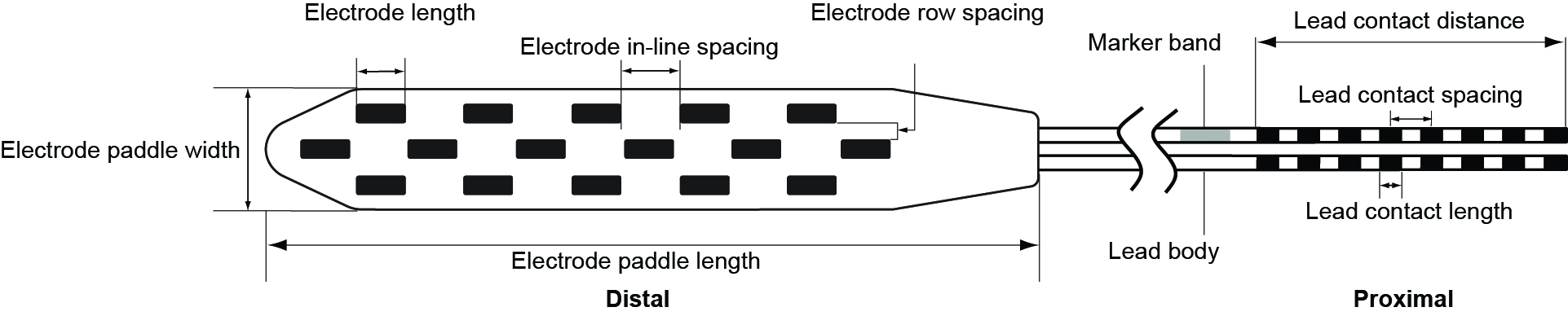


The above SCS system is indicated as an aid in the management of chronic, intractable pain of the trunk and/or limbs. The first release of the SCS system entered the commercial market in the USA as a result of PMA840001 on November 30, 1984. SCS received CE mark approval in 1993.
