# Supplementary material for: Spinal cord stimulation for predominant low back pain in failed back surgery syndrome: study protocol for an international multicenter randomized controlled trial (PROMISE study)
Source: Trials. 2013 Nov 7;14:376. doi: 10.1186/1745-6215-14-376 (PMC4226255; doi:10.1186/1745-6215-14-376)
Supplement: Additional file 2 — Is a table presenting the Site Ethics/IRB approvals (as of 3 July 2013). [file 1745-6215-14-376-S2.docx]

**Additional file 2. Site Ethics/IRB approvals (as of 3^rd^ July 2013)**

| **Site name** | **Principal Investigator name** | **Ethics committees (EC)/Institutional Review Board (IRB) name** | **EC/IRB Street Address** |
| --- | --- | --- | --- |
| Centre Hospitalier Régional de la Citadelle | Remacle Jean Michel H.A. | Comité d’éthique - CHR La Citadelle | Boulevard du 12e Ligne 1 |
| GZA - Sint Augustinus ziekenhuis | Van Havenbergh Tony | Ethische commissie GZA - Sint Augustinus ziekenhuis | Oosterveldlaan 24 |
| H-Hartziekenhuis Roeselare-Menen vzw | Deruytter Marc | Medisch-Ethische Commissie - Heilige Hart Ziekenhuis Roeselare-Menen | Wilgenstraat 2, Pijn klinik, Westlaan 123 |
| Cliniques Universitaires Saint-Luc | Raftopoulos Christian | Comité d'éthique - Cliniques Universitaires Saint-Luc | Avenue Hippocrate 55.14, Comission d'éthique Biomédicale Hopitalo-Facultaire, Tour Harvey |
| Algemeen Ziekenhuis Sint Maarten - Campus Rooienberg | Vangeneugden johan | Ethisch Commissie - Emmaüs vzw | Edgard Tinellaan 1c |
| Centre Hospitalier Universitaire de Poitiers - Hôpital de la Milétrie | Rigoard Phillippe | Comité de Protection des Personnes "Ouest III" - CHU de Poitiers | 2 rue de la Milétrie, Pavillon Le Blaye - Porte 9 |
| Städtisches Klinikum Görlitz gGmbH | Eif Marcus | Sächsische Landesärztekammer | Schützenhöhe 16 |
| Sint-Elisabeth Ziekenhuis | Van Eijs Franciscus | Medisch Ethische Toetsingscommissie - Sint-Elisabeth Ziekenhuis | Hilvarenbeekseweg 60, Postbus 90151 |
| Hospital Clínico Universitario de Valladolid | Noriega David | Comité Ético de Investigación Clínica - Hospital Clínico Universitario de Valladolid | Avda. Ramón y Cajal, 7, Farmacología 6º Planta, Facultad de Medicina |
| Hospital Clinico Universitario de Valencia | Garcia March Guillermo | Comité Ético de Investigación Clínica - Hospital Clínico Universitario de Valencia | Avda. Blasco Ibañez 17, Pabellón B-primer piso |
| Oxford University Hospitals NHS Trust - John Radcliffe Hospital | Bojanic Stana | NRES Committee Yorkshire and The Humber - Sheffield | First Floor, Millside, Mill Pond Lane, Meanwood |
| Queens Medical Centre | Basu Surajit | NRES Committee Yorkshire and The Humber - Sheffield | First Floor, Millside, Mill Pond Lane, Meanwood |
| National Pain Institute | Sajan Cherian | Florida Hospital IRB | 212 E Winter Park St |
| Richmond Bone and Joint Clinic | Burnette Candice | WIRB (Western Institutional Review Board) | 3535 7th Ave SW |
| New York Spine and Wellness Center | Calkins Anne | Saint Joseph's Hospital Health Center Research Committee IRB | 301 Prospect Ave |
| Pain Care LLC | Galan Vincent | WIRB (Western Institutional Review Board) | PO BOX 12029 |
| West Virginia University Pain Management Center | Kim Chong | West Virginia University Office of Research Compliance | 886 Chestnut Ridge Rd, PO Box 6845 |
| Wellspan Interventional Pain Management | Vu To-Nhu | WellSpan Health IRB | 1001 S George St |
| Kozmary Center for Pain Management | Kozmary Steven | WIRB (Western Institutional Review Board) | 3535 7th Ave SW |
| Ocean Springs Neuroscience Center | Edmiston Bart | WIRB (Western Institutional Review Board) | 3535 7th Ave SW |
